# Wave optics of imaging with contact ball lenses

**DOI:** 10.1038/s41598-023-32826-8

**Published:** 2023-04-24

**Authors:** A. V. Maslov, B. Jin, V. N. Astratov

**Affiliations:** 1 Department of Radiophysics, University of Nizhny Novgorod, Nizhny Novgorod, 603022 Russia; 2 Department of Physics and Optical Science, University of North Carolina at Charlotte, Charlotte, NC 28233-0001 USA

**Keywords:** Sub-wavelength optics, Micro-optics, Imaging and sensing, Super-resolution microscopy, Nanophotonics and plasmonics, Sub-wavelength optics

## Abstract

Recent progress in microspherical superlens nanoscopy raises a fundamental question about the transition from super-resolution properties of mesoscale microspheres, which can provide a subwavelength resolution $\sim \lambda /7$, to macroscale ball lenses, for which the imaging quality degrades because of aberrations. To address this question, this work develops a theory describing the imaging by contact ball lenses with diameters $30<D/\lambda <4000$ covering this transition range and for a broad range of refractive indices $1.3<n<2.1$. Starting from geometrical optics we subsequently proceed to an exact numerical solution of the Maxwell equations explaining virtual and real image formation as well as magnification *M* and resolution near the critical index $n\approx 2$ which is of interest for applications demanding the highest *M* such as cellphone microscopy. The wave effects manifest themselves in a strong dependence of the image plane position and magnification on $D/\lambda$, for which a simple analytical formula is derived. It is demonstrated that a subwavelength resolution is achievable at $D/\lambda \lesssim 1400$. The theory explains the results of experimental contact-ball imaging. The understanding of the physical mechanisms of image formation revealed in this study creates a basis for developing applications of contact ball lenses in cellphone-based microscopy.

## Introduction

The development of compact, portable, and light-weight optical detection and imaging devices requires the use of microoptics, including millimeter and sub-millimeter size lenses. Historically, the construction of single lens microscopes, in particular with ball lenses, by Antonie van Leeuwenhoek in the 17-th century enabled him to discover microorganisms establishing a new direction of life sciences^1–3^. Although later in the 18-th and, especially, the 19-th centuries microscopy evolved mainly in favor of compound microscopes operating with multi-lens objectives and bulky stands, the interest in compact microoptics solutions, including single ball lens designs, was revitalized in the 1990s and 2000s due to the advent of megapixel CCD and CMOS sensor arrays used in the microscope cameras. The efforts to use uncorrected minilenses (including balls) as a part of optical systems run into fundamental obstacles because the resulting images become affected by various aberrations. Optical aberrations, in general, put the practical limit on the performance of various optical systems^4^. For example, spherical aberration leads to blurry images and focus errors which are well known in photography. Aberrations exacerbate imaging quality when working with subjects at close distances and using lenses with high-curvature surfaces or wide apertures. Yet, these are essentially the operating conditions of microoptical imaging.

On the other hand, a novel type of microscope imaging based on placing microspheres in direct contact with nanoscale objects has emerged in the last decade and has been termed “microsphere-assisted” or “microsphere superlens” imaging (MSI)^5–21^. It has been demonstrated experimentally that such microspheres create magnified virtual images with resolution $\sim \lambda /7$ well beyond the classical diffraction limit. These results generated a significant interest in the mechanisms of such imaging because of its label-free nature, inherent simplicity, and potential biomedical applications. Such an extraordinarily successful application of wavelength-scale microspheres raises a question about the role of aberrations in MSI. Indeed, a spherical lens is expected to give rise to aberrations and, for example, lens systems in cellphone cameras rely on aspherical lenses to achieve high quality imaging^22^. Moreover, the applicability of the concept of aberrations to microspheres is not well justified due to the fact that the optical operation of such contact microspheres cannot be viewed as a result of some rather small deviation from an ideal case as in the classical geometrical optics (GO). The involvement of objects’ near-fields often in a form of plasmonic or localized surface plasmon resonance excitation and extreme curvature of the wavelength-scale microspheres call for an exact solution of the Maxwell equations as the only possible way of theoretical understanding of such imaging^23–30^. The exact numerical solutions predicted the resolution at $\lambda /4-\lambda /5$ approaching the experimental values. This area remains an active field of theoretical studies where such factors as structured illumination with the plasmonic hot spots^20^, coherent contributions^26^, enhancement of near-fields under coupling with whispering gallery modes (WGMs) in microspheres^27^, and extreme curvature of the wavelength-scale microspheres^23^ are being considered to explain experimentally observed super-resolution values.

**Figure 1:**
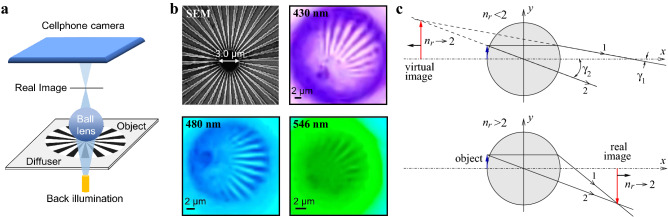
Experimental measurements and imaging regimes. (**a**) Schematic of the experimental imaging through a ball lens using a cellphone. (**b**) Scanning electron microscopy (SEM) image of a Siemens star and its cellphone camera images through the ball (LASFN35 glass, $R=1$ mm) using optical filters at the specified wavelengths. (**c**) Geometrical optics of imaging with a ball lens for an object at the surface. A virtual image is created for $n_r<2$ and a real image for $n_r>2$. The black arrows indicate the displacement of the image as $n_r\rightarrow 2$.

The purpose of this work can be seen as filling the gap between the classical imaging approach based on geometrical optics with aberrations included in analysis for sufficiently large ball lenses on the one hand and a limiting case of contact, wavelength-scale microspheres for which introducing aberrations is conceptually difficult on the other hand. We focus specifically on objects near the optical axis where the spherical aberration and defocus aberration dominate. We consider the imaging by ball lenses with the index of refraction $1.3<n<2.1$ and radius $15<R/\lambda <2000$ which are placed in contact with the objects. Such imaging can be viewed as an extension of MSI towards using submillimeter- and millimeter-scale ball lenses which are much more convenient for developing portable and lightweight imaging systems not requiring bulky and expensive microscope objectives with heavy microscope stands. In addition, millimeter-scale ball lenses provide larger field-of-view compared to microspheres. The perfect example of such a system is a cellphone operating in combination with a contact ball lens^31^ illustrated in Fig. 1a. The interest to similar systems operating with noncontact ball lenses emerged in the last decade^32–37^. However, some problems such as insufficient magnification, spherical aberration, and pincushion distortions were found to be limiting factors for developing this technology with the best resolution about 1.5 $\upmu$m reported up to date.

In our theoretical analysis we make a gradual transition from the paraxial to nonparaxial ray tracing and, finally, to wave optics (WO) based on a numerical solution of the Maxwell equations which provide an accurate description of imaging by the contact ball lenses with various refractive indices and sizes. As expected, our WO results converge to that of GO for larger ball lenses, while they also show a large number of novel properties taking place in the intermediate range of sizes. Using a two-dimensional (2-D) model, we study the effect of focal shift for contact ball lenses for the first time. It is shown that it plays a significantly larger role for real imaging taking place for $1.9<n<2.1$ in the size range $50<R/\lambda <2000$, compared to the virtual imaging observed for $1.3<n<1.9$. We also show that the effect of focal shift becomes more significant for smaller ball lenses leading to a dramatic reduction of the image magnification compared to the GO predictions. It is shown that the resolution improves for smaller ball lenses reaching deeply subwavelength value $\sim \lambda /2$ at $R/\lambda \lesssim 100$ in the vicinity of $n\approx 2$. The index variation about $n\approx 2$ affects greatly the magnification but not the resolution. Using diffraction integrals we clarify the role of defocus and spherical aberration on the shift of the image plane and also derive a simple formula describing the focal shift in a wide range of parameters $\lambda$, *n*, and *R* in a good agreement with the exact numerical solution of the Maxwell equations. We show that the WO results for the image plane shift agree well with the experimental cellphone microscopy of a Siemens star through a contact $R=1$ mm ball lens at three different values of its refractive index. Finally, we experimentally demonstrate that cellphone microscopy based on using ball lenses with *n* sufficiently close to the critical index $n\approx 2$ allows reaching $\sim 0.9$ $\upmu$m resolution values in a resonable agreement with theoretical predictions.

## Geometrical optics of ball lenses

### Geometry and typical experimental situation

The use of ball lenses with the index of refraction $n\approx 2$ can provide very large magnification^31^. The experimental setup for imaging through a ball lens is shown in Fig. 1a. The measurement details are described in Methods. The imaged object was a Siemens star and the real images were recorded by a cellphone camera, see Fig. 1b. The variation of the refractive index near $n\approx 2$ was obtained due to the material dispersion of the glass and the selection of the operating wavelength using optical filters. The measured magnification can be very high, in the range 20–50, but depends strongly on the refractive index, see Table 1. The image analysis also showed that the magnification *M* depends linearly $M=x_i/R$ on the distance $x_i$ between the image plane and the center of the ball.

**Table 1:** Comparison of the experimental measurements (EM) of image location $x_i/R$ for a ball with $R=1$ mm at different operating wavelength $\lambda$ and the predictions for $x_i/R$ of various theoretical approaches based on the refractive indices *n* of LASNF35 glass at the specified wavelengths: paraxial geometrical optics (PGO), numerical wave optics (NWO), and simple analytical result (10) obtained from WO (AWO).

$\lambda$ (nm)	*n*	$R/{\lambda }$	$x_i/R$ (EM)	$x_i/R$ (PGO)	$x_i/R$ (NWO)	$x_i/R$ (AWO)
430	2.068	2326	26	30.4	25.0	25.0
480	2.049	2083	35	41.8	31.9	31.8
546	2.030	1832	46	67.7	43.4	43.6

### Paraxial geometrical optics

Let us now analyze the experimental results on the ball-lens imaging within the framework of paraxial GO. The application of GO is rather natural here since the size of the ball is much larger than the wavelength. We consider a ball with radius *R* and refractive index $n_2$ surrounded by medium with $n_1$, see Fig. 1c. An object is located next to the surface of the ball so that the gap is significantly smaller than the wavelength. The variation of the gap *g*(*y*) due to curvature can be estimated as $g(y)=y^2/(2R)$, where *y* is the displacement from the optical axis. Assuming a subwavelength condition $g<\lambda /2$ gives the maximum object size $L=2\sqrt{R\lambda }$ for which the subwavelength gap approximation holds. Taking, for example, $R=1$ mm and $\lambda =0.5$ $\upmu$m yields $L\approx 40$ $\upmu$m. Thus, the subwavelength gap approximation can easily hold for various samples of interest, such as used in the experiments, see Fig. 1b. Thus, the first interface does not participate in the image formation. The emission diagram formed inside the lens is transformed by the refraction at the second interface. The curvature of the first interface, however, can play a role in increasing the resolution due to the outcoupling of evanescent fields but this is likely to take place for lenses with sizes in the micron range. Ray 1, which originates from a point at distance $h\ll R$ from the optical *x* axis and propagates parallel to it, upon refraction bends at an angle $\gamma _1=(n_r-1)h/R$, where $n_r=n_2/n_1$, while ray 2, which passes through the center of the ball, has $\gamma _2=h/R$. The equations for the two refracted rays

1 $$\begin{aligned} y_1(x)=-(n_r-1)(h/R)(x-R)+h,\quad y_2(x)=-(h/R)x \end{aligned}$$

allow finding their intersection $x_i$ and resulting object magnification *M*:

2 $$\begin{aligned} x_i/R = -M, \quad M=n_r/(2-n_r). \end{aligned}$$

This equation was also used to evaluate magnification in virtual imaging^10,24^ and later generalized to the case of a finite gap between the object and the ball lens^12^. The intersection is virtual ($x_i<0$) if $\gamma _1<\gamma _2$ or $n_r<2$ and real ($x_i>0$) in the opposite case. According to Eq. (2), the distance $|x_i|$ and magnification *M* increase rapidly as $n_r\rightarrow 2$. Since $M\propto x_i$, we can limit ourselves to finding the image location $x_i$ for a point source on the optical axis.

The image plane positions $x_i/R$ predicted by the paraxial GO for the experimental parameters using Eq. (2) are significantly larger than the measured values, see Table 1. Moreover, the difference increases drastically as $n_r$ approaches 2. In general, the paraxial approximation is applicable only if the image is formed by rays in a small angular range, often limited by apertures in practice. The failure of the paraxial GO can be explained by the fact that the imaging here was performed using the full angular range offered by the ball lens.

### Ray tracing beyond the paraxial approximation

**Figure 2:**
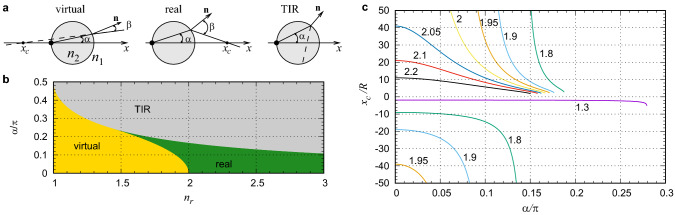
Nonparaxial ray tracing for a point source at the surface of a ball lens. (**a**) As the emission angle $\alpha$ increases, the intersection $x_c$ of the refracted ray with the optical axis can change from virtual to real and then disappear in the TIR case. (**b**) Propagation regimes for the refracted ray at various $n_r=n_2/n_1$ and $\alpha$. (**c**) Virtual and real intersections $x_c/R$ versus $\alpha$ for various values of $n_r$ shown next to the curves.

Let us now go beyond the paraxial approximation and consider various rays originating from a point source located at $x=-R$, Fig. 2a. A ray emitted at an angle $\alpha$ is refracted at the interface. The angle of refraction $\beta$ is determined by Snell’s law: $n_2\sin \alpha =n_1\sin \beta$. The refracted ray either crosses the *x* axis at some $x_c$ or disappears in the case of total internal reflection (TIR) if $\alpha >\arcsin (n_1/n_2)$. The intersection can be virtual ($x_c<0$ at $\beta <2\alpha$) or real ($x_c>0$ at $\beta >2\alpha$). The regimes are determined by $n_r$ and $\alpha$, see Fig. 2b. At $1<n_r<\sqrt{2}$ only virtual intersections exist. At $\sqrt{2}<n_r<2$ there are real and virtual intersections. At $n_r>2$ there are only real intersections.

Ideal images (virtual or real) are formed if all refracted rays intersect at the same point $x_i=x_c$ giving $M=-x_i/R$. In practice, $x_c$ depends on $\alpha$:

3 $$\begin{aligned} \frac{x_c}{R} = \sin (2\alpha )\left( \frac{1}{\tan (\beta -2\alpha )}+\frac{1}{\tan (2\alpha )}\right) , \end{aligned}$$

see Fig. 2c. In the limit $\alpha \rightarrow 0$ one can recover from (3) the paraxial approximation (2). The variation of $x_c(\alpha )$ near the focus for paraxial rays $x_c(\alpha \rightarrow 0)$ is referred to as spherical aberration. For rather small $n_r$, see $n_r=1.3$ in Fig. 2c, $x_c$ is negative and depends on $\alpha$ very weakly and, therefore, a sharp virtual image is expected to be formed. At $n_r=1.8, 1.9, 1.95$, the virtual image position $|x_c|$ increases and its variation with $\alpha$ becomes very significant. This results in image blurring and, probably, disappearance. Moreover, at $n_r>\sqrt{2}$ one obtains $x_c\rightarrow -\infty$ as $\alpha \rightarrow \alpha ^{*}$, with $\alpha ^{*}=\arccos (n_2/(2n_1))<\pi /4$. When $\alpha$ exceeds $\alpha ^{*}$, the intersection $x_c$ becomes real and strongly dependent on $\alpha$. At $n_r=2.05, 2.1, 2.2$ the dependence on $\alpha$ becomes flatter and that should increase the sharpness of the real image. At $n_r=2$ there is no intersection with *x*, i.e., $x_c\rightarrow \infty$ as $\alpha \rightarrow 0$.

Thus, the rays emitted from the point source at $x=-R$ do not converge to a well defined spot for $1.9\lesssim n_r\lesssim 2.2$. Under the severely strong spherical aberration the appearance of well resolved images is unlikely. Yet, the experiments show distinct images, see Fig. 1b. Thus, the formation of images and their locations observed experimentally seem to be in disagreement with the GO predictions.

## Wave optics of ball lenses

### Theoretical model

**Figure 3:**
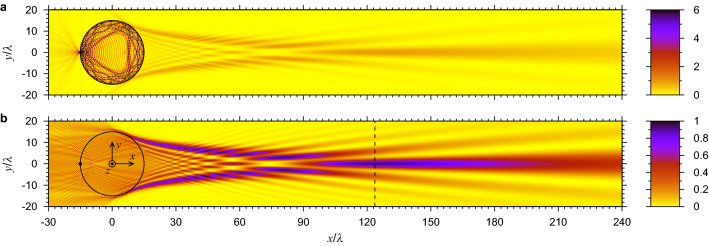
Point source emission near a ball with $R/\lambda =15$, $n_2=2$ located inside $n_1=1$: (**a**) $|E_z(x,y)|^2$ and (**b**) ${\text{psf}}(x,y)$. The distributions in both frames are normalized to the maximum of ${\text{psf}}(x,y)$ at $x/\lambda =124$, $y=0$. In frame (**b**) the dot shows the position of the point source and the dashed vertical line at $x/\lambda =124$ defines the optimal image plane (diffraction focus).

To explain the image formation let us now adopt the wave theory. We consider a 2-D model, in which the fields depend on *x* and *y* only, and take a point current source $J_z(x,y)=j_0\delta (x-R)\delta (y)$, see the coordinate system in Fig. 3b. Without the ball, the same point source located at $x=y=0$ generates in the uniform medium an outgoing cylindrical wave with the electric field $E_z^u(x,y)=-\pi E_0 H_0^{(1)}(k_1\rho )$, where $H_0^{(1)}$ is the Hankel function, $\rho =\sqrt{x^2+y^2}$, $k_1=n_1\omega /c$, and $E_0=j_0\omega /c^2$. Note that $|E_z^u(k_1\rho )|=E_0$ at the distance $\rho /\lambda \approx 0.9969/n_1$ from the source so that $E_0$ can be conveniently used for normalization. The fields can be calculated without any further approximations by solving the Maxwell equations using the expansion into the cylindrical functions^23,26,27,38^. In general, modeling rigorously optical structures with sizes of hundreds and thousands of wavelengths thick is very challenging. Often this requires adopting various wave-propagation methods^39^ which rely on some approximations, such as neglecting back-propagating fields. In the diffraction theory the field intensity is also represented as an integral over some aperture with a kernel which depends on the fields^40^. Since the fields at the aperture are not known, various assumptions are made, such as taking the incident field only (the Kirchhoff’s approximation)^41,42^. The specific circular geometry of the present problem allows finding the fields with accuracy limited only by computational precision rather than by a priori assumptions. The comparison of 2-D and 3-D FDTD results of modeling the focal distances of 10–18 $\upmu$m-diameter spheres shows only a small difference while all trends remain essentially the same^43^. Thus, the application of a 2-D model here for significantly larger spheres is quite rational from the computational point of view and its results are expected to hold also in 3-D.

In the simulations we take a vacuum background $n_1=1$ and various values for $n_2$. The results for $n_1\ne 1$ can be obtained directly from those for $n_1=1$ as it follows from the Maxwell equations. Indeed, let us assume that we have some arbitrary current that produces fields in two situations, (a) and (b), so that the spatial dependences of the refractive index differ by a constant factor *s*: $n_b(\textbf{r}) = s\;n_a(\textbf{r})$. The fields in case (b) are related to that in (a) as $E_b(\textbf{r}, \omega )=E_a(\textbf{r}, s\omega )/s$ and $H_b(\textbf{r}, \omega )=H_a(r, s\omega )$. Thus, the case of a background index $n_1>1$ is equivalent to the case with $n_1=1$ but with operation at a proportionately higher frequency or, equivalently, shorter wavelength. This also increases the resolution by the same factor as in immersion microscopy.

### Image plane position and magnification

The spatial spectrum of the fields in the far-field region from the ball at $x\gg R$ defines the image (real and virtual) which is formed by the objective (or cellphone camera) lens, see Fig. 1a. The image can be found by recreating the intensity in the focal plane of the objective lens using the far fields only^23,24,26,44,45^. This is equivalent to backpropagating the fields from some plane $x\gg R$ to the image plane. The distribution of the image intensity along *y* for a given location of the focal plane *x* gives the 2-D point spread function (PSF) ${\text{psf}}(x,y)=|E_z^b(x,y)|^2$, where $E_z^b(x,y)$ is the backpropagated field. The backpropagated field $E_z^b(x,y)$ coincides with the true field $E_z(x,y)$ at distances larger than a few wavelengths from the ball at $x>R$. In the other regions they differ from one another. In particular, the true field diverges at the location of the point source while the backpropagated field (and PSF) does not. Note that without the ball, the *z*-polarized current point source located at the origin $x=y=0$ produces far fields that after backpropagation give at $x=0$ the image intensity ${\text{psf}}(0,y)=\pi ^2E_0^2J_0^2(k_1y)$, where $J_0$ is the Bessel function. This implies the angular range of $-\pi /2<\varphi <\pi /2$ for the collection of the outgoing waves. This image has the first zero at $y/\lambda = 2.4/(2\pi n_1)=0.38/n_1$ and its full width at half maximum (FWHM) is $W/\lambda =0.36/n_1$. So the *z*-polarized source gives a smaller width compared to a *y*-polarized one^23^ which can be attributed to its uniform angular emission.

To understand the role of wave phenomena in image formation we take a ball with $R/\lambda =15$ ($kR=94.25$) and $n_2=2$, see Fig. 3. While its size is smaller than in the experiments shown in Fig. 1a, b it is still much larger than the wavelength. The paraxial GO predicts no ray convergence in this case, see Eq. (2). The intensity $|E_z(x,y)|^2$ diverges near the point source and its value exceeds the color scale range in Fig. 3a. The intensity $|E_z(x,y)|^2$ inside the ball near its boundary is also very high because of TIR. This accumulation of energy takes place even without any resonances, such as WGMs. The WGMs can also exist but their quality factor is extremely high around $R/\lambda =15$ with $n_2=2$ so that their excitation requires extremely fine tuning of $R/\lambda$ or $n_2$. The ${\text{psf}}(x,y)$ in Fig. 3b coincides with the true intensity $|E_z(x,y)|^2$ in Fig. 3a for $x>R$ but differs substantially for $x<R$. In particular, ${\text{psf}}(x,y)$ has no enhancement inside the ball or singularity near the point source. The peak at $x/\lambda =124$ is the diffraction focus which is formed in contrast to the lack of ray convergence in the paraxial GO. Its location can be considered as the image plane $x_i$. The longitudinal width of the peak (or the focusing depth) is very large, $\gtrsim 20\lambda$. However, the transverse width is very small. Indeed, the FWHM of the central peak is $W=3.42\lambda$. Taking into account the magnification $M=124/15=8.27$, the resolution becomes $W/M=0.41\lambda$, which is close to the PSF width of $0.36\lambda$ obtained in free space in 2-D for a *z*-polarized point source under the condition of light collection in the largest possible angular range of $-\pi /2<\varphi <\pi /2$. Thus, the ball itself does not produce any significant broadening of the PSF.

**Figure 4:**
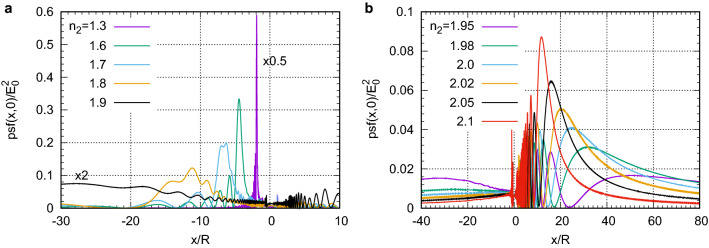
Dependence of ${\text{psf}}(x,0)/E_0^2$ for a ball with $R/\lambda =100$ ($kR=628$) and a point source at $x=-R$ for various values of $n_2$. The formation of (**a**) virtual and (**b**) real images is shown. In frame (**a**), the curves for $n_2=1.3$ and $n_2=1.9$ were multiplied by the indicated factors.

Having established the presence of focusing enabled by the contact ball with $R/\lambda =15$, let us now move on to larger balls, closer to the GO regime. To investigate the location of the image plane we plot in Fig. 4 the PSF along the optical axis as the refractive index of the ball $n_2$ changes from 1.3 to 2.1 at a fixed size $R/\lambda =100$. At $n_2=1.3$ a sharp peak is observed at $x/R=-1.91$ corresponding to the location of the virtual image, see Fig. 4a. As $n_2$ increases, the peak rapidly becomes smaller and broader. At $n_2=1.9$, a noticeable intensity also appears at $x/R>0$. At $n_2=1.95$ the furthest maximum of intensity is at $x/R\approx 50$, however, the second one at $x/R\approx 15$ is higher, see Fig. 4b. As $n_2$ increases even more, the furthest maximum becomes narrower, higher, and closer to the ball. The furthest peak is still slightly smaller than the second peak at $n_2=1.98$ but it overcomes it at $n_2=2$. Note that ${\text{psf}}(x,0)$ always oscillates along *x*. These oscillations can disappear in practice if imaging is performed using illumination with a broad spectrum. However, the slow variation can still remain if the illumination bandwidth is sufficiently small. The plane locations at the maxima along *x* that differ from the furthest one cannot produce images because the PSF has much stronger sidelobes in the transverse *y* direction, see, for example, the plane at the second maximum at $x/\lambda =56$ in Fig. 3b. Thus, the furthest peak defines the image plane location $x_i$.

**Figure 5:**
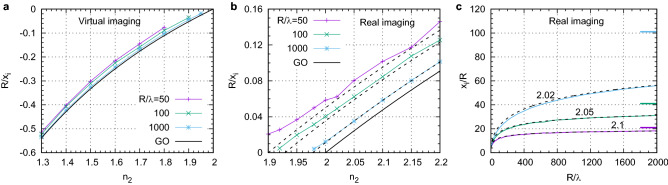
Dependence of the image plane position $x_i$, which is defined by the maximum of ${\text{psf}}(x,0)$, on $n_2$ and $R/{\lambda }$. The corresponding magnification is $M=-x_i/R$. (**a,b**) Inverse of $x_i$ as a function of $n_2$ for several $R/\lambda$. The dashed lines running next to the solid lines in (**b**) are the corresponding analytical results given by Eq. (10). (**c**) $x_i/R$ as a function of $R/\lambda$ for several $n_2$ indicated near the curves. The horizontal lines on the right side show the corresponding GO values of *M* given by Eq. (2). The dashed black lines show the analytical results described by Eq. (10).

We can now compare the predictions of GO with that of WO. Due to symmetry of the problem, *M* in both cases is directly related to the image location $M=-x_i/R$. Since $x_i$ and *M* diverge as $n_r\rightarrow 2$ in the GO limit, it is convenient to analyze graphically $R/x_i=-1/M$. Figure 5a,b show the comparison for virtual and real imaging, respectively, for several ball sizes: $R/\lambda =$50, 100, and 1000. For virtual images, WO predicts slightly larger $|x_i|/R$ (or smaller $R/|x_i|$) as compared to that from GO. However the agreement with GO becomes closer as $R/\lambda$ increases. More interesting situation is observed for real images. The function $R/x_i$ for all $R/\lambda$ behaves linearly with $n_2$ having practically the same slope as predicted by GO. However, the lines are shifted and the shift depends on $R/\lambda$. With increasing $R/\lambda$, the shift decreases and eventually one recovers the GO regime. The presence of even a small shift in $R/x_i$ in the region of $n_r\approx 2$ (or $1/M\approx 0$) can lead to dramatic deviations of $x_i/R$ and *M* from the GO predictions. The image plane positions calculated using WO for the indices $n_2$ and sizes $R/\lambda$ realized in the experiments are given in Table 1 and agree well with the experimental measurements.

Figure 5c shows magnification (and therefore, the position of the real image plane $x_i=|M|R$) as a function of $R/\lambda$ for $n_2=2.02$, 2.05, and 2.1. In all cases |*M*| initially grows with $R/\lambda$. If $n_2$ differs from 2 significantly, for example, $n_2=2.1$, then |*M*| saturates quite rapidly at $R/\lambda \sim 200-300$. If the difference $n_2-2$ becomes smaller, then the growth of |*M*| becomes much slower and reaching the asymptotic GO values requires much larger values of $R/\lambda$. For $n_2=2.05$, for example, |*M*| still continues growing slightly even for $R/\lambda \sim 1000$. For $n_2=2.02$, the growth of |*M*| is very pronounced even for $R/\lambda \sim 2000$. Thus, the convergence to the GO regime is determined not only by the ratio $R/\lambda$ but also by the difference $n_2-2$. When the difference $n_2-2$ is small, the deviation from the paraxial GO even for large lenses remains very significant. This conclusion is supported well by the experimental results, see Table 1.

### Resolution

**Figure 6:**
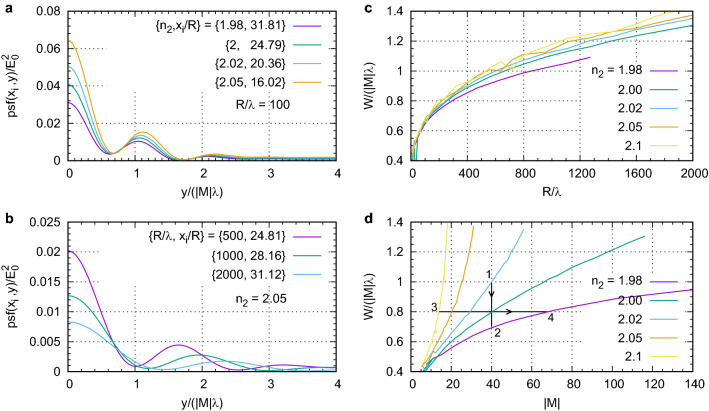
Resolution of ball lens imaging. (**a**) ${\text{psf}}(x_i,y)$ as a function of the transverse coordinate *y* at the positions $x_i$ of the diffraction focus for several values of $n_2$ and fixed $R/\lambda$. (**b**) same as (**a**) but for several values of $R/\lambda$ and fixed $n_2$. (**c**) Resolution $W/(|M|\lambda )$ as a function of $R/\lambda$ for several values of $n_2$. (**d**) Resolution $W/(|M|\lambda )$ as a function of magnification $|M|=x_i/R$ for several values of $n_2$.

According to the Houston criterion the resolution is defined as the FWHM of the PSF^46^. After finding the image of the point source, the width *W* of the central peak was divided by |*M*| and normalized to $\lambda$. Figure 6a shows the transverse dependence of ${\text{psf}}(x_i,y)$ at the image plane $x_i$ for various $n_2$ and fixed $R/\lambda =100$. In all cases the PSF has sidelobes. Their height relative to the central peaks decreases as $n_2$ increases from 1.98 to 2.05. In the estimates of the FWHM of the PSF we only consider the central peak keeping in mind the adverse effect of large sidelobes. The FWHM of the central peak does not change significantly: $W/|M|=0.64\lambda$ at $n_2=1.98$ and $W/|M|=0.67\lambda$ at $n_2=2.05$. Although this resolution slightly worse than the diffraction limit $\lambda /2$, it is surprisingly high considering the large spherical aberration, see Fig. 2c. Thus, for a fixed $R/\lambda$ the resolution does not change significantly for different $n_2$ but magnification at $n_2=1.98$ is larger than that at $n_2=2.05$. On the other hand, the larger sidelobes at $n_2=1.98$ will lead to lower image quality. The resolution decreases with increasing $R/\lambda$. For example, in Fig. 6b the FWHM increases from $W/(|M|\lambda )=0.98$ at $R/\lambda =500$ to $W/(|M|\lambda )=1.38$ at $R/\lambda =2000$.

Figure 6c shows that the PSF width $W/(|M|\lambda )$ for a fixed $n_2$ grows with increasing $R/\lambda$ but does not significantly depend on $n_2$. On the other hand, the magnification |*M*| increases both with $n_2$ and $R/\lambda$, see Fig. 5c. Thus, for a fixed $n_2$ the increase of |*M*| always comes at the expense of decreasing resolution. This trend is illustrated in Fig. 6d which shows the relation between the resolution and magnification for several $n_2$. A natural question arises of whether we can achieve simultaneously high resolution (small PSF width) and large magnification. Unfortunately, the answer to this question is negative. Indeed, let us fix a rather high $|M|=40$ and try to increase the resolution by moving in the direction $1\rightarrow 2$ indicated in Fig. 6d. The apparent increase in resolution here is accompanied by the growth of the sidelobes. The same growth takes place if we try to fix resolution, $W/(|M|\lambda )$=0.8, and move in the direction $3\rightarrow 4$ in attempt to increase magnification.

### Analytical results

Having the exact WO solution at our disposal, let us now try to build an approximate model which would yield a simpler physical interpretation of the image formation. In our specific case the fields are expanded into the cylindrical functions and the expansion coefficients are subsequently calculated. Let us now represent the field at any arbitrary (observation) point $\textbf{r}$ as an integral over the surface just outside the ball:

4 $$\begin{aligned} E_z(\textbf{r}) = \int \limits _0^{2\pi }\!\!{\text{d}}\varphi '\;E_z(\textbf{r}, \varphi '), \quad E_z(\textbf{r}, \varphi ') = R\left( G^{e}_{zz}(\textbf{r}, \mathbf{r'}) J_z^e(\mathbf{r'}) + G^{m}_{z{\varphi }}(\textbf{r}, \mathbf{r'}) J^m_{\varphi }(\textbf{r}')\right) , \end{aligned}$$

where $\textbf{r}'$ is a point at the cylindrical surface with radius *R*. In Eq. (4), the effective electric and magnetic currents, which are obtained from the fields outside the cylinder^47^, and the Green’s functions are

5 $$\begin{aligned} J_z^e=\frac{c}{4\pi }H_{\varphi },\quad J_{\varphi }^m=\frac{c}{4\pi }E_z,\quad G^{e}_{zz} = -\frac{\pi \omega }{c^2}H_0^{(1)}(k_1 s), \quad G^{m}_{z\varphi }=in_1\frac{\pi \omega }{c^2}H_1^{(1)}(k_1s)\frac{\textbf{s}\cdot \mathbf{r'}}{sr'},\quad \textbf{s}=\textbf{r}-\mathbf{r'}, \end{aligned}$$

where $H_{0,1}^{(1)}$ are the Hankel functions. To investigate the contribution to $E_z(\textbf{r})$ from different parts of the integration surface, let us define the cumulative field, which is formed by the currents in the angular range limited by $[-\varphi :\varphi ]$,

6 $$\begin{aligned} E_z^c(\textbf{r}, \varphi ) = \int \limits _{-\varphi }^{\varphi }\!\!{\text{d}}\varphi '\;E_z(\textbf{r}, \varphi '), \end{aligned}$$

so that the integral over the full circle $\varphi =\pi$ gives the actual field at the observation point: $E_z(\textbf{r})=E_z^c(\textbf{r}, \pi )$. Note that the field is calculated using the integration over a closed surface, not a finite opening in a screen which is common in the diffraction theory.

**Figure 7:**
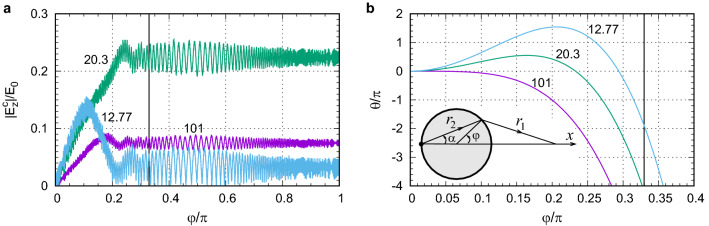
(**a**) Cumulative field $|E^c_z(x, \varphi )|$ for $R/\lambda =100$ at several observation points: $x/R=101$ (focus according to paraxial GO), $x/R=20.3$ (diffraction focus defined by the furthest PSF maximum), $x/R=12.77$ (PSF minimum). (**b**) Phase difference $\theta (\varphi )$, see Eq. (7), between the ray initially propagating at $\alpha$ (and crossing the surface at $\varphi =2\alpha$) and the axial ray for the same observations points as in (**a**). For both frames $n_r=2.02$. The vertical solid lines in (**a**) and (**b**) define the TIR angle $\varphi =2{\text{arcsin}}(1/2.02)\approx 0.33\pi$.

Figure 7a shows the magnitude of the cumulative field $|E_z^c(x, \varphi )|$ as a function of $\varphi$ for $n_2=2.02$ at several special locations on the *x* axis: $x/R=101$ (GO focus), $x/R=20.3$ (diffraction focus), and $x/R=12.77$ (the furthest minimum of PSF). At the GO focus, the contributions $|E_z^c(\textbf{r}, \varphi )|$ originate from a rather small angular range $\varphi /\pi \lesssim 0.15$. At the diffraction focus, the contributions increase more rapidly, come from a larger interval $\varphi /\pi \lesssim 0.25$, and subsequently result in a much larger value for $|E_z(\textbf{r})|$. At the PSF minimum, the contributions initially increase and then start to decrease leading eventually to a very small $|E_z(\textbf{r})|$.

The rigorous representation of the field at any observation point using the fields at the circle just outside the ball, see Eq. (4), can also be recast into a simplified form. We can assume that the effective currents are approximately the same in amplitudes but their phases are determined by the distance from the source to the location on the circle. The contributions of the currents can also be assumed to vary only in phase. The field at an observation point becomes dependent only on the optical path difference $\theta$ between an off-axis path and the axial one, see the inset in Fig. 7b,

$$\begin{aligned} E_z(\textbf{r}) \sim \int \!\!{\text{d}}\alpha \;\;e^{i\theta (\alpha )}, \end{aligned}$$

where

7 $$\begin{aligned} \theta (\alpha ) = kn_2 (r_2-2R) + kn_1(r_1 -d) = 2 n_2 kR(\cos \alpha -1)+ n_1kd\left( \sqrt{1+4(R/d)(1+R/d)\sin ^2\alpha }-1\right) \end{aligned}$$

and $d=x_i-R$. For $\alpha \ll 1$ we obtain

8 $$\begin{aligned} \theta (\alpha ) \approx n_1 kR \left( f_2\alpha ^2+f_4\alpha ^4\right) ,\; f_2 = 2-n_r+\xi ,\; f_4 = -\left( 8-n_r+16\xi +12\xi ^2+3\xi ^3\right) /12,\; \xi =2R/d. \end{aligned}$$

The term $f_2$ describes defocus aberration and $f_4$ describes spherical aberration^48^. By setting $f_2=0$ in Eq. (8) we can obtain the focal distance $d_f=2R/(n_r-2)$ and magnification in the paraxial GO approximation (2).

The focusing properties of an optical systems are often characterized by the Fresnel number *N* of the exit pupil with radius *R*: $N=R^2/(\lambda d_f)$, where $d_f$ is the GO focal distance. Typically, the presence of a sharp focus requires $N\gg 10$. For $N\lesssim 10$, the defocus tolerance can be significant. Moreover, the maximum of irradiance can shift closer to the exit pupil. The regime $N\lesssim 10$ can significantly differ from the predictions of GO due to the influence of diffraction which defines not only the resolution but also the position of maximum intensity. In the case of ball lenses, there is no clearly defined aperture. However, to estimate the Fresnel number we can simply use the ball radius *R* since the effective aperture is formed by the TIR:

9 $$\begin{aligned} N\approx \frac{n_1R^2}{d_f\lambda }=\frac{n_1(n_r-2)}{2}\frac{R}{\lambda }. \end{aligned}$$

For large $d_f$, which are obtained for small $n_r-2$, the Fresnel number becomes small. For example, the Fresnel number reaches $N=10$ only at $R/\lambda =1000$ if $n_r=2.02$. As seen from Fig. 5c, at $R/\lambda =1000$ the focal position is still significantly smaller than the GO prediction.

The phase difference allows us to explain the shift of the intensity maximum as compared to the paraxial GO. Figure 7b shows the phase difference $\theta (\alpha )$ as a function of the location on the circle $\varphi =2\alpha$ for the same observation points on the *x* axis as in Fig. 7a. In general, if the spherical aberration term vanishes $f_4=0$, the focal point is defined by $f_2=0$ and its intensity is determined by the integral over the full aperture (limited by TIR) resulting in the largest possible intensity. The deviation $f_2\ne 0$ from the GO focus leads to the blurring of the image due to defocus aberration. The spherical aberration $f_4\ne 0$, see the line for $x/R=101$ in Fig. 7b, gives rise to a rapid phase decrease for $\alpha /\pi >0.15$ and therefore, to the oscillations of the integrand reducing significantly its value. A larger intensity can be obtained if one moves away from the GO predicted focus in such a way as to provide the smallest variation of the phase $\theta (\alpha )$ over the aperture. Since usually $f_4<0$, compensating it requires $f_2>0$, and therefore, taking smaller *d* as compared to the paraxial GO prediction. Indeed, at the point of maximum, $x/R=20.3$ in Fig. 7b, the phase deviation from zero is limited to $|\theta |\lesssim \pi /2$ in the interval $\varphi /\pi <0.25$. For even smaller *x*, the minimum at $x/R=12.77$ in Fig. 7b, the phase changes significantly bringing the total intensity to almost zero.

We can further apply this simple physical picture to derive an analytical curve that describes the image plane location and magnification. From Fig. 7b we can note that at the location of intensity maximum the phase difference is limited to $\theta \lesssim \pi /2$. Indeed, exceeding this value would cancel out the contributions from the smaller angles. Thus, we can state that at the extremum $\theta (\alpha ^*)=\pi /2$. Using (8) we can find $\alpha ^*$ and then relation between $f_2$ and $f_4$. Further, we can approximate $f_4\approx -1/2$ and obtain

10 $$\begin{aligned} M=-\frac{x_i}{R}\approx \frac{n_r}{n_r-2+\delta },\quad \delta =\sqrt{\frac{\pi }{n_1kR}}=\sqrt{\frac{\lambda }{2n_1R}}. \end{aligned}$$

The paraxial GO result described by Eq. (2) follows from the more general Eq. (10) in the limit $R/\lambda \rightarrow \infty$. The WO effects in the position of the image plane and corresponding magnification are accounted for by the extra term $\delta$, which depends on the size parameter *kR*. Note that one can often use GO formulas and account for the effects not described by GO by introducing some effective parameters, for example, an effective refractive index of a spherical lens which becomes a function of its diameter^43^. In our case, we obtained the GO result from the more general WO result and, therefore, we do not need to resort to any effective parameters.

Figure 5c shows that simple formula (10) describes the fully numerical results very accurately. This extra term also agrees with the *kR*-dependent shifts of the straight lines in Fig. 5b, especially for large $R/\lambda$. Moreover, Eq. (10) explains accurately the experimentally measured image plane locations, see Table 1. The analytical derivation of Eq. (10) uses the phase difference between the path characterized by $\alpha \ne 0$ and that for $\alpha =0$. We neglected the variation of intensity as a function of $\alpha$ in order to obtain a simple result which, however, agrees well with the fully numerical modeling. In the 3-D case, the formula for the optical path difference remains valid but one may need to account for the solid angle factor $\sin \alpha$ and specific orientation of the emitter.

We can conclude that there are two main physical factors that contribute to the shift of the image plane in contact-ball imaging. First, even an aberration-free converging spherical front does not focus to its origin once an aperture is introduced^41,42^. The focal point moves towards the aperture and the shift increases with decrease of the Fresnel number of the aperture. When the relative refractive index of the ball is $n_r\approx 2$, the geometric focus goes to infinity and, therefore, the Fresnel number decreases drastically. Second, in the case of contact ball lenses the phase front immediately after the ball suffers from spherical aberration and, therefore, it cannot focus to a single point. In terms of ray optics, the spherical aberration is particularly significant near $n_r\approx 2$ when no single focal point for the rays refracted by the ball can be defined. Both factors define the distribution of the diffracted intensity in contact-ball imaging. Within the realm of WO the intensity at a given point can be written as a diffraction integral over an aperture effectively defined by TIR of the spherically aberrated wavefront. The axial intensity reaches a maximum at the point at which the defocus and spherical aberration terms, see Eq. (8), can partially compensate each other over the integration aperture.

### Experimental resolution

**Figure 8:**
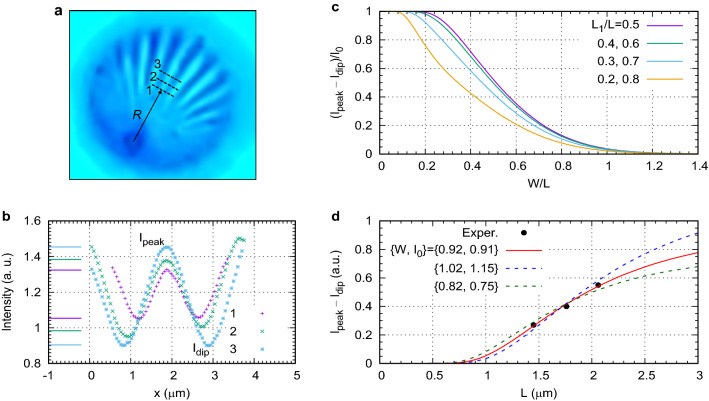
Quantification of resolution provided by a ball lens with $R=1$ mm at $\lambda$=480 nm. (**a**) Image of the Siemens star showing the cross-sections (dashed lines) along which the intensity profiles were measured. (**b**) Experimental intensity profiles (points) and estimated maximum and minimum of intensity levels (horizontal solid lines). The *x* scale is obtained after dividing the image coordinates by magnification. (**c**) Theoretical variation $f(W/L)=(I_{\rm peak}-I_{\rm dip})/I_0$ for a periodic stripe pattern for several values of $L_1/L$. (**d**) Experimental points fitted with the theoretical *f*(*W*/*L*) at $L_1/L=0.6$ for several sets of FWHM *W* and intensity $I_0$. The solid red line shows the best fit while the two dashed lines show much worse fits for slightly different parameter sets.

The resolution of the ball lens imaging can also be estimated from the experiments shown in Fig. 1a,b. The image of the Siemens star was scanned at several distances from the center, see Fig. 8a,b. The metallic spokes manifest themselves as the dips. The oscillations of the intensity become larger as the local period of the structure increases. The image intensity contains some spatially uniform background level I_b_ due to various scattering in the experimental setup. Using the SEM of the Siemens star, see Fig. 1b, the gap-to-pitch ratio $L_1/L$ can be estimated as 0.6 in the area of the scans.

To extract the resolution from the measurements it was assumed that at each scan the star can be described as a 1-D structure in which each period *L* contains a bright stripe with intensity $I_0$ and width $L_1$. The image is obtained by the convolution of this ideal object with a Gaussians PSF defined by its FWHM *W*. According to the Houston resolution criterion^46^, the FWHM of the PSF represents the resolution of the system. The resultant image is also a periodic structure which can be characterized by the intensity at the peak $I_{\rm peak}$ and at the dip $I_{\rm dip}$. For a specific geometrical parameter $L_1/L$, the intensity depends only on the ratio *W*/*L* and therefore, one can define

11 $$\begin{aligned} I_{\rm peak}-I_{\rm dip}=I_0f(W/L), \end{aligned}$$

where $0<f(W/L)<1$. The difference $I_{\rm peak}-I_{\rm dip}$ does not depend on the presence of the background level $I_{\rm b}$ in the measurements and, therefore, can be directly used for fitting once the geometric function *f*(*W*/*L*) is known. Figure 8c shows the function *f*(*W*/*L*) at several values of $L_1/L$. Note that this function is symmetric relative to $L_1/L=0.5$, that is it takes the same value, for example, for $L_1/L=0.4$ and $L_1/L=0.6$. In all cases the modulation amplitude *f*(*W*/*L*) decreases with increasing *W*/*L*. The oscillations become observable for $W/L\lesssim 1$.

The fitting of the analytical dependence (11) with $L_1/L=0.6$ to the experimental points, see Fig. 8d, gives $W=0.92$ $\upmu$m or $W/\lambda =1.9$. Varying *W* near this optimal value makes the fit worse and therefore, the approach allows a reliable extraction of resolution. Indeed, the curve for $W=1.02$ $\upmu$m cannot be fitted to the points better than shown since any variation of $I_0$ would only scale the curve up or down and therefore, worsen the fit. The value $W/\lambda =1.9$ is slightly greater than the PSF obtained in the simulations $W/(|M|\lambda )$=1.4, see the curve for $n_2=2.05$ at $R/\lambda \approx 2000$ in Fig. 6c. The knowledge of $I_0$ allows estimating the background level $I_{\rm b}$ as well. Without the background scattering and for small periods the intensity should oscillate symmetrically around its average value $(L_1/L)I_0=0.6I_0=0.55$ while the average value for scan 1 is $I_{\rm ave}=1.19$. This gives the background level $I_{\rm b}=I_{\rm ave}-0.6I_0=0.64$. Thus, the background scattering is very significant and should be accounted for in resolution estimations.

We also note that for a gold double-stripe object the resolution was previously estimated^31^ to be $W=0.65$ $\upmu$m at $\lambda =480$ nm or $W/\lambda =1.4$. This resolution is better than in the present study of the star. The difference can be attributed to several factors. One factor is related to geometry since the double-stripe element of the long-period array in Ref.^31^ is practically an isolated object and, therefore, is more suitable for resolution quantification^49^ compared to the star with azimuthally periodic spokes, which also have a width gradient in the radial direction. Another factor is related to different reflection properties of thin metallic layers, Au in Ref.^31^ versus Cr in the present study.

## Conclusion

Despite a tremendous interest in MSI methods developed with wavelength-scale microspheres^5–21^, the connection of this field of studies to the standard wave theory of aberrated imaging by macroscopic ball lenses has not been previously established. In this work we developed a comprehensive approach to this problem for ball lenses with diameters varying from $D/\lambda \approx 30$ (quite often used in MSI studies) up to $D/\lambda \approx 4000$ (reaching the limit of millimeter-scale ball lenses). Our approach is based on the transition from geometrical optics to full-wave solutions of the Maxwell equations. A unique feature of our numerical modeling approach is that it bridges wave phenomena taking place at completely different spatial scales – the near-field coupling of a point source, the field propagation inside ball lenses with diameters $30\lesssim D/\lambda \lesssim 4000$, and, finally, the formation of the diffracted field at distances $\sim 10^5\lambda$.

Our theory is developed specifically for the case of a direct contact of a ball lens with nanoscale objects with intention to increase the resolution due to near-field effects in a spirit of the solid immersion lens concept^50,51^. Another feature of the theory is its ability to describe accurately the imaging by ball lenses with refractive index near the critical value $n\approx 2$, for which the deviations from geometrical optics become dramatic. This critical regime is very attractive for imaging applications because of extremely large $M>50$ magnification^31^. We showed that the image plane location and magnification in this regime can be described correctly only by wave optics which predicts a significant shift of the image plane towards the ball with corresponding reduction in magnification, in accord with the presented experimental evidence. The shift becomes more significant as ball’s size becomes smaller and *n* closer to 2. This effect is governed mostly by diffraction since the Fresnel number of the effective aperture is small. It is demonstrated that the exact location of the image plane is defined by the counterplay of defocusing and spherical aberrations. This allowed us to derive a simple correction to the geometric formula for the image plane location. In contrast to common expectation, we showed that the resolution improves for smaller ball lenses reaching deeply subwavelength values $\sim \lambda /2$ at $R/\lambda \lesssim 100$ in the vicinity of $n\approx 2$. The index variation about $n\approx 2$ affects greatly the magnification but not the resolution.

Although the dispersion $n(\lambda )$ is present in all microspheres used previously for MSI, its impact on imaging becomes dramatic only in the vicinity of the critical value $n\approx 2$ for contact microspheres in the air environment studied here. These effects are significantly less pronounced for the relative indices $1.4<n<1.8$ which are typical for the previously studied silica microspheres in air or BTG microspheres in water, in PDMS, or in plastic environments.

To experimentally test our theoretical predictions, we performed a resolution quantitation aimed at the demonstration of potential advantages of ball lenses with the specially designed index for cellphone-based microscopy applications. Currently, the resolution of such portable and lightweight microscopes is pixel-limited at a $\sim 1.5$-$\upmu$m level due to insufficient magnification. Using a Siemens star object imaged through a LASFN35 glass ball lens with $n=2.049$ at $\lambda =480$ nm we demonstrated a resolution of $\sim 0.9$ $\upmu$m or $1.9\lambda$, slightly below the resolution of $1.4\lambda$ predicted by our theory, and magnification $M\approx 35$. In our previous studies we approached the wavelength-scale resolution using similar approaches^31^. Thus, this experiment demonstrates a potential of the proposed methods for increasing the resolution of imaging based on using contact ball lenses with $n\approx 2$. The peculiar imaging properties of contact ball lenses with $n\approx 2$ complement their ability to focus plane waves to strongly enhanced photonic nanojets on the outer edge^52^. The theoretical methods developed in this work demonstrate a gradual transition from MSI methods to classical imaging by aberrated macroscopic ball lenses with applications in high-resolution cellphone-based microscopy.

## Methods

### Experiments

All experiments were performed using the cellphone-based transmitted light microscopy shown in Fig. 1a. The illumination from a tungsten halogen lamp was provided through narrow (about 10 nm) bandpass filters with transmission peaked at different wavelengths. A white light 120-grit ground glass diffuser (Edmund Optics) was installed at 8-mm distance below the sample to provide widefield incoherent illumination with a broad range of incident angles. The object was a Siemens star (Edmund Optics) made of 36 equidistant Cr spokes on a silica substrate. The ball lens was made of LASFN35 glass with the refractive index varying from about 2 to 2.1 as the wavelength changes from 700 nm to 400 nm. The measurements were performed in air environment. The optical images in Figs. 1b and Fig. 8a were recorded using a cell phone camera (Samsung Galaxy S9+). The SEM image in Fig. 1b was obtained using the Raith 150 e-beam lithography system. The location of the scans in Fig. 8a were selected close to the central part of the image to minimize pincushion distortion. The center of the Siemens star was shifted to the edge of the limited circular field-of-view where all distances are locally distorted. The local coordinate scale along lines (1–3) in Fig. 8a was determined by the magnification data independently obtained for the same ball lens at the central part of the image using a double-stripe object with known physical dimensions.

### Simulations

All simulations were performed using in-house developed codes in C programming language.

## Data Availability

The data that support the findings of this study are available from the corresponding author upon reasonable request.
